# Circulating Cell-Free DNA Captures the Intratumor Heterogeneity in Multinodular Hepatocellular Carcinoma

**DOI:** 10.1200/PO.21.00335

**Published:** 2022-03-09

**Authors:** Mairene Coto-Llerena, Andrej Benjak, John Gallon, Marie-Anne Meier, Tuyana Boldanova, Luigi M. Terracciano, Charlotte K.Y. Ng, Salvatore Piscuoglio

**Affiliations:** ^1^ Institute of Medical Genetics and Pathology, University Hospital Basel, Basel, Switzerland; ^2^ Visceral Surgery and Precision Medicine Research Laboratory, Department of Biomedicine, University of Basel, Basel, Switzerland; ^3^ Department for BioMedical Research (DBMR), University of Bern, Bern, Switzerland; ^4^ Hepatology Laboratory, Department of Biomedicine, University of Basel, Basel, Switzerland; ^5^ Division of Gastroenterology and Hepatology, University Hospital Basel, Basel, Switzerland; ^6^ Department of Anatomic Pathology, IRCCS Humanitas Research Hospital, Rozzano, Milan, Italy; ^7^ Department of Biomedical Sciences, Humanitas University, Pieve Emanuele, Milan, Italy; ^8^ SIB Swiss Institute of Bioinformatics, Lausanne, Switzerland

## Abstract

Hepatocellular carcinoma (HCC) is a highly heterogeneous disease, with more than 40% of patients initially diagnosed with multinodular HCCs. Although circulating cell-free DNA (cfDNA) has been shown to effectively detect somatic mutations, little is known about its utility to capture intratumor heterogeneity in patients with multinodular HCC undergoing systemic treatment.

**MATERIALS AND METHODS:**

Tumor biopsies and plasma were synchronously collected from seven prospectively recruited patients with HCC before and during systemic therapy. Plasma-derived cfDNA and matched germline were subjected to high-depth targeted sequencing with molecular barcoding. The mutational profile of the cfDNA was compared with whole-exome sequencing from matched tumor biopsies.

**RESULTS:**

Genomic data revealed that out of the seven patients, five were considered intrahepatic metastasis and two multicentric HCCs. cfDNA captured the majority of mutations in the tumors and detected significantly more mutations than tumor biopsies. Driver mutations such as *CTNNB1* S33C, *NRAS* Q61R, *ARID1A* R727fs, and *NF1* E2368fs as well as standard-of-care biomarkers of response to targeted therapy were detected only in cfDNA. In the two patients with multicentric HCC, cfDNA detected mutations derived from the genetically independent and spatially distinct nodules. Moreover, cfDNA was not only able to capture clonal mutations but also the subclonal mutations detected in only one of the multiple biopsied nodules. Furthermore, serial cfDNA detected variants of tumor origin emerging during treatment.

**CONCLUSION:**

This study revealed that the genetic analysis of cfDNA captures the intratumor heterogeneity in multinodular HCC highlighting the potential for cfDNA as a sensitive and noninvasive tool for precision medicine.

## INTRODUCTION

Hepatocellular carcinoma (HCC), the most common primary liver cancer, is the third leading cause of cancer-related deaths.^1^ In patients with advanced HCC, where chemoembolization is not appropriate or has failed, systemic therapy is the only available option.^2^ One major challenge in the treatment of HCC is high tumor heterogeneity because of the existence of multinodular HCCs, and the high intratumor molecular diversity.^3^ Approximately 41% to 75% of patients are initially diagnosed with multinodular HCCs, which limits the access to curative treatment options and leads to poor prognosis.^4-6^ Multinodular HCCs represent intrahepatic metastasis (IM) of a single cancer or multicentric occurrence (MC).^7,8^ Whole-exome sequencing (WES) analysis of paired and recurrent HCC showed that IM pairs shared 15%-93% of mutations, whereas MC pairs only 0%-0.28%.^7^ The high tumor heterogeneity, especially in multicentric HCC, assessed from the view of a single tumor biopsy can lead to underestimation of the genetic heterogeneity of the tumor and could present a major challenge to precision medicine and biomarker development.

**CONTEXT**

**Key Objective**
More than 40% of hepatocellular carcinomas (HCCs) are initially diagnosed with multinodular HCCs. Since HCC is usually diagnosed without liver biopsy and performing multiple liver biopsies on multinodular disease is typically clinically unfeasible, we sought to investigate the utility of cell-free DNA (cfDNA) in capturing the intratumor heterogeneity in patients with multinodular HCC.
**Knowledge Generated**
Most mutations detected in the tumors were also detected in the cfDNA, including both interlesion heterogeneity in multicentric HCC and intratumor heterogeneity in intrahepatic metastases. Temporal heterogeneity in response to treatment can be captured by serial cfDNA samples.
**Relevance**
cfDNA reflects the genomic landscape of multicentric HCC and HCCs with intrahepatic metastases. Given the limited access to tumor tissues, especially in spatially distinct regions of multinodular HCCs, our results highlight the potential of cfDNA as a noninvasive tool for tumor monitoring and genomic profiling.

The use of circulating tumor DNA in place of biopsies for the genetic profiling of HCC may offer an advantage in capturing tumor heterogeneity. Tumor-derived DNA is shed into the bloodstream by necrotic and apoptotic tumor cells. Studies have demonstrated the use of cell-free DNA (cfDNA) as an alternative to liver biopsies for molecular profiling.^9,10^ Recently, it has also been shown that cfDNA can be used to identify predictive biomarkers of response to systemic therapies in HCC.^11^ However, the ability of cfDNA to capture tumor heterogeneity in multinodular HCC has not been evaluated. Here, we sought to evaluate the potential of cfDNA to capture tumor heterogeneity in patients with multinodular HCC undergoing systemic therapy.

## MATERIALS AND METHODS

### Patients and Samples

Seven patients diagnosed with multinodular HCC at the University Hospital Basel (Basel, Switzerland) were included. All patients received sorafenib (Nexavar). Patients P2 and P9 received nivolumab (Opdivo) as a second line of treatment. Radiologic (according to mRECIST criteria^12,13^) and biochemical follow-up were used to monitor treatment response. The first set of ultrasound-guided biopsies were diagnostic liver biopsies,^9,14^ and the second biopsy was taken 2 weeks after treatment initiation (sorafenib). Upon tumor progression, a follow-up tumoral biopsy was performed. In some patients, a biopsy was taken before the initiation of a second round of treatment (sorafenib or nivolumab). Synchronous to biopsy collection, whole blood was collected for cfDNA extraction (Data Supplement).

### Sequencing Analysis of Circulating Free DNA and Biopsies

cfDNA and germline DNA were subjected to targeted sequencing covering the exonic regions of 75 genes recurrently mutated in HCC and *TERT* promoter using unique molecular identifiers (UMI) technology (Cell3 Target; Nonacus Ltd, Birmingham, United Kingdom, Data Supplement). Sequencing was performed to a mean consensus read depth of 1775X on Novaseq 6000 (Data Supplement). Somatic variants were detected using Mageri^15^ and UMI-VarCal^16^ (Data Supplement).

DNA from the HCCs and paired nontumor biopsies were subjected to WES (Data Supplement).^14^ Somatic mutations were detected using Mutect2 (GATK 4.1.4.1)^17^ and Strelka2 v2.9.10^18^ (Data Supplement). Clonal reconstruction was performed using ABSOLUTE (v1.0.6)^19^ and PhylogicNDT^20^ (Data Supplement).

Written informed consent was obtained from all patients. The study was approved by the ethics committee of the Northwestern part of Switzerland (Protocol Number EKNZ 2014-099).

## RESULTS

### cfDNA As a Surrogate for the Genetic Profiling of Multinodular HCC

**FIG 1.**
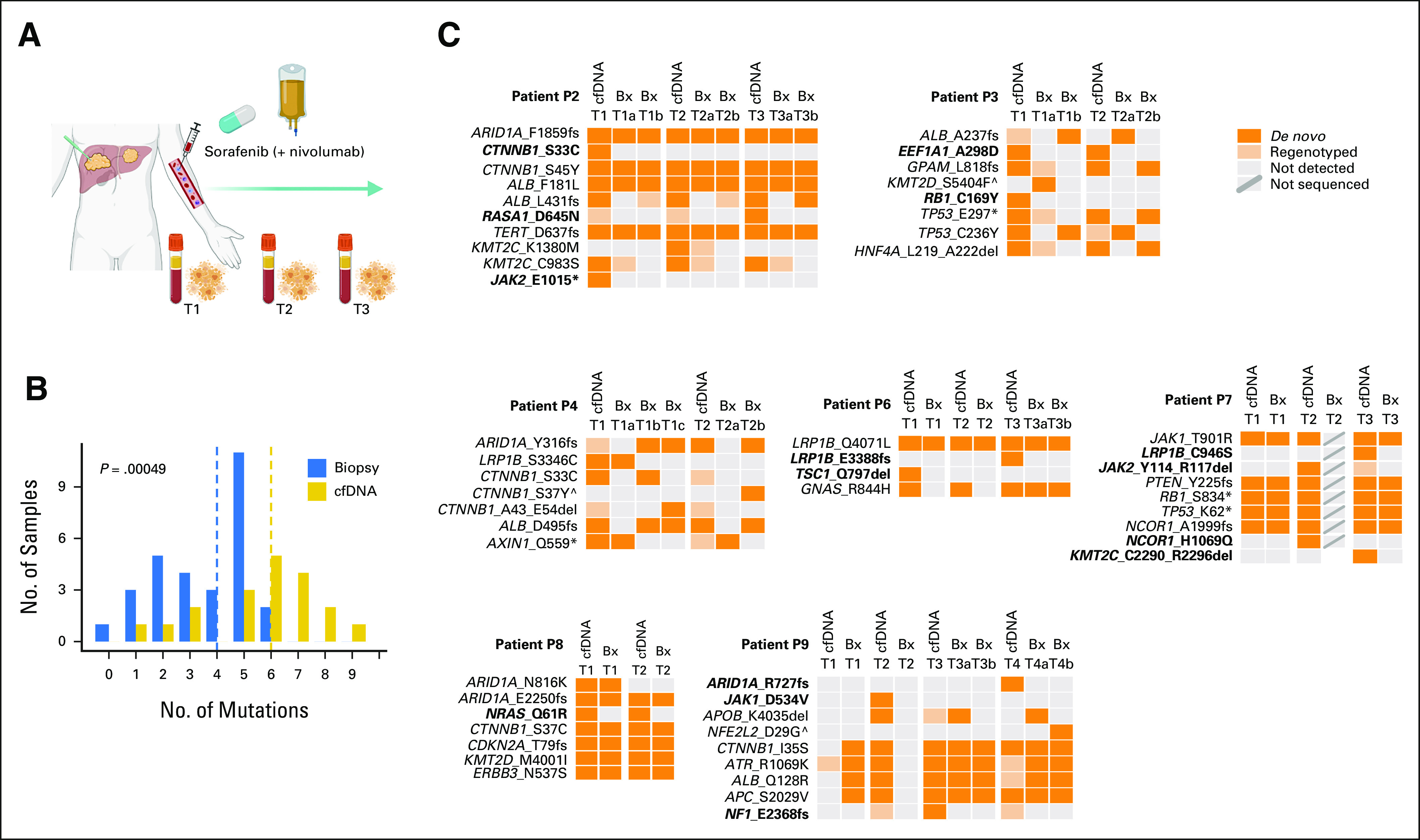
cfDNA as a surrogate for the genetic profiling of multinodular hepatocellular carcinoma. (A) Schematic representation of the serial collection of cfDNA and tumor biopsies during the clinical course. (B) Histogram showing frequency of nonsynonymous mutations (detected by de novo calling or by regenotyping) detected in cfDNA (yellow) and tumor biopsy (blue). The vertical dashed lines indicate the medians. Statistical analysis was performed using Mann-Whitney U test. (C) Comparison of nonsynonymous mutations detected in cfDNA (N = 19) versus tumor biopsies (N = 27). Samples are grouped by patient and organized by time point. Somatic mutations are color-coded according to the legend. Dark orange boxes indicate de novo detected mutations; light orange boxes indicate mutations detected by regenotyping guided by other cfDNA or tumor biopsy samples from the same patient; gray boxes indicate undetected mutation and gray boxes with a diagonal line indicate samples that were not sequenced. Mutations in bold indicate those that were detected only in cfDNA samples. Mutations including ^ symbol indicate those detected only in the biopsies. Created with BioRender.com. cfDNA, cell-free DNA; P, patient.

To determine the ability of cfDNA to detect somatic mutations in multinodular HCC, we collected plasma-derived cfDNA (n = 19) and one or more tumor biopsies from seven prospectively recruited patients with HCC before the initiation of sorafenib, during, and/or after sorafenib treatment (Fig 1A, Table 1, Data Supplement). Two of the patients received nivolumab as a second line of treatment. The average concentration of cfDNA was 24.5 ng/mL of plasma (range, 3.9-206 ng/mL, Data Supplement).

**TABLE 1.**
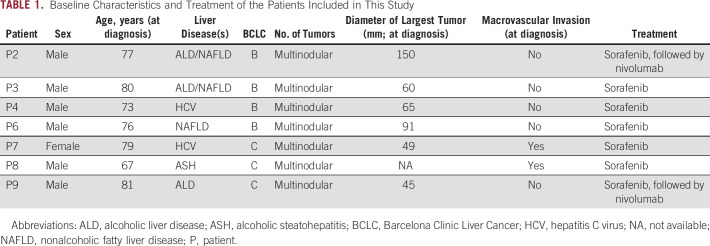
Baseline Characteristics and Treatment of the Patients Included in This Study

We sequenced the cfDNA and their peripheral blood mononuclear cell–derived normal counterparts using a custom UMI-tagged assay targeting the exonic regions of 75 genes commonly altered in HCC and *TERT* promoter, and the tumor biopsies using WES (n = 29, Fig 1A and Data Supplement). We identified more mutations in cfDNA (median, 6; range, 0-9) than tumor biopsies (median, 4; range, 0-6; *P* = .00049, Mann-Whitney U test; Fig 1B). Of the 80 mutations detected in at least one biopsy, 72 (90%) were detected in the synchronous cfDNA, 5 (6%) in at least one cfDNA at another time point, and 3 (4%) were not detected in any other sample (Fig 1C). 23/25 *CTNNB1/TP53/ARID1A* mutations were detected in the synchronous cfDNA. Notably, no mutation was detected in tumors at multiple time points but consistently not detected in any cfDNA.

Across the cfDNA samples (except P7 T2, for which tumor biopsy was not sequenced), 101 mutations were detected. *CTNNB1*/*TP53*/*ARID1A* mutations were observed in most cfDNA samples, with 13 *CTNNB1* mutations detected in 10 cfDNA samples (P2, P4, P8, and P9), nine *ARID1A* mutations in eight cfDNA (P2, P4, P8, and P9), and seven *TP53* mutations in five cfDNA (P3 and P7; Fig 1C). 72/101 (71%) were detected in at least one synchronously collected tumor biopsy, 9/101 (9%) in at least one tumor biopsy at another time point, 11/101 (11%) were not detected in any tumor biopsy but were detected in at least one other cfDNA sample, and 9/101 (9%) were not detected in any other sample (Fig 1C). Most mutations detected in the cfDNA and at least one synchronous tumor biopsy were de novo detected (ie, not regenotyped, given the mutations present in other samples of the same patient) in the cfDNA (63/72; 84%). Of the nine cfDNA mutations detected in at least one tumor biopsy at another time point, five came from P9 T2, at which no mutation was detected in the tumor in part because of its low tumor purity. The remaining four likely constitute genuine tumor-derived variants below detection limits in the synchronous tumor samples. Finally, the 20 cfDNA-specific variants that were detected in at least one other cfDNA or no other sample included likely HCC driver mutations, including *CTNNB1* S33C (P2), *NRAS* Q61R (P8), and *ARID1A* R727fs and *NF1* E2368fs (both P9).

To determine the factors that may influence the detectability of a mutation in the cfDNA, we compared molecular and clinicopathological parameters between mutations detected and not detected in the cfDNA. Variants not detected in the cfDNA are associated with lower variant allele frequency (VAF) of the corresponding variants in the matched tissues (*P* = .0071, Mann-Whitney U test; Data Supplement), suggesting that some low-frequency mutations in tumor subclones may exist in the cfDNA but are below the detection level of the current assay. Additionally, those mutations not detected de novo in the cfDNA were present in smaller tumors compared with mutations detected de novo in the cfDNA (*P* = .0067, Mann-Whitney U test; Data Supplement). Other clinicopathologic parameters such as necrosis and alpha-fetoprotein were not associated with the detection of mutations in the cfDNA (Data Supplement).

Taken together, our results demonstrate that nearly all mutations identified in the tumor biopsies could be detected in the cfDNA, whereas approximately 20% of the mutations identified in the cfDNA could not be identified by WES of the tumor biopsies.

### cfDNA Captures Interlesion Heterogeneity in Multicentric HCC

**FIG 2.**
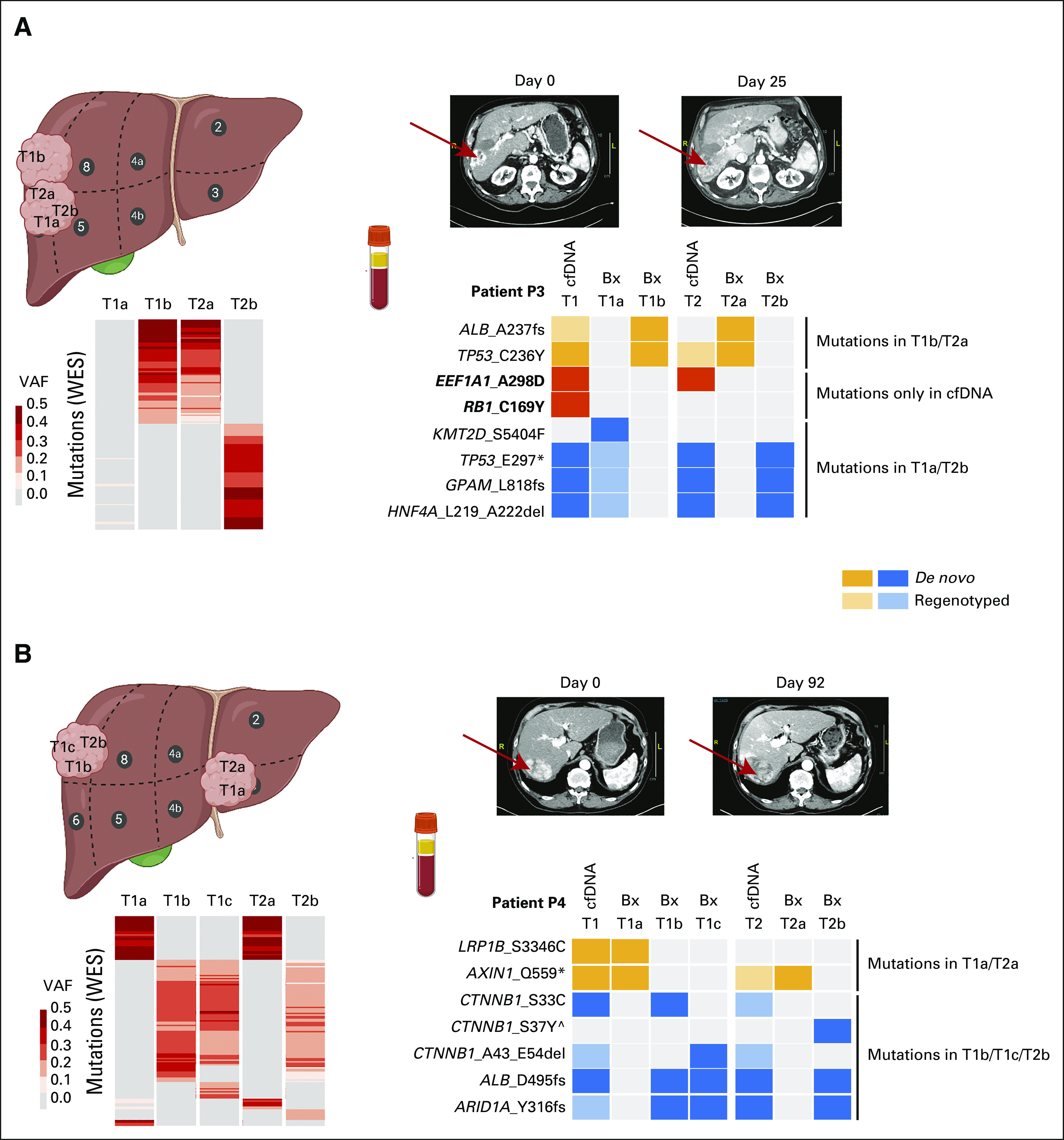
cfDNA captures interlesion heterogeneity in multicentric HCC. Schematic representation of two patients, (A) P3 and (B) P4, with multicentric HCC showing the location of the biopsies taken (top left), and the radiologic images at different time points (top right). Somatic mutations detected by WES in the tumor biopsies are shown in the bottom left panel, with their VAFs color-coded according to the legend. The variants identified in biopsies and cfDNA in the regions covered by the targeted sequencing panel are shown in the bottom right. Mutations detected in the tumors are color-coded in blue or orange according to the independent primary tumors, further indicated as de novo (dark) or regenotyped (light). Mutations in bold and red indicate those that were detected only in cfDNA samples. Mutations including ^ symbol indicate those detected only in the biopsies. Note: sample T1a from P3 had low tumor purity. Created with BioRender.com. cfDNA, cell-free DNA; HCC, hepatocellular carcinoma; VAF, variant allele frequency; WES, whole-exome sequencing; P, patient.

Multicentric HCCs arise from the development of independent primary tumors, and would thus display genetically distinct profiles.^7^ WES analysis of the tumor biopsies showed that P3 and P4 had multicentric disease (Fig 2 and Data Supplement). We therefore asked whether cfDNA would capture the mutations harbored in the individual primary tumors.

P3 was treated with sorafenib for 1 month before tumor progression (Fig 2A top-right). Two pretreatment biopsies (T1a, segment 6; and T1b, segment 7) and two other biopsies while the patient was on sorafenib (T2a and T2b, both segment 6, Fig 2A top-left and Data Supplement) were taken. Histopathologic analysis confirmed T1a and T2b as HCC, whereas T1b and T2a were mixed HCC-cholangiocarcinoma. WES revealed that T1a/T2b and T1b/T2a did not share any somatic mutations and had different *TP53* mutations (C236Y and E297*), indicating the genetic independence of these pairs (Fig 2A bottom and Data Supplement). Before treatment initiation (T1), cfDNA captured the 3/4 mutations in T1a and 2/2 mutations in T1b, demonstrating that cfDNA can capture mutations from the distinct primary tumors. Similarly, under sorafenib treatment (T2), 1/2 mutation in T2a and 3/3 mutations in T2b were captured in the cfDNA sample (Fig 2A bottom-right). Additionally, *EEF1A* A298D and *RB1* C169Y mutations were detected in the cfDNA and absent in both tumors, suggesting that these mutations might either be present in another region of these nodules or in a nonbiopsied nodule. There was no correlation between the VAFs in the cfDNA and in the tumors (*R*^2^ = 0.12, *P* > .05, Data Supplement).

P4 was treated with sorafenib for 3 months with a 1-month interval because of adverse effects and had progressive disease (Fig 2B top-right). Five biopsies were taken before (T1) and during (T2) sorafenib treatment. T1a and T2a were from segment 3, and T1b, T1c, and T2b were from segment 7 (Fig 2B top-left and Data Supplement). WES showed that the biopsies from segments 3 and 7 did not share any somatic mutation (Fig 2B bottom and Data Supplement). Interestingly, the three segment 7 biopsies (T1b, T1c, and T2b) all had distinct *CTNNB1* mutations (S33C, S37Y, and A43_E54del). Here, the pretreatment cfDNA (T1) captured 2/2 mutations seen in T1a and 4/4 mutations seen in T1b/T1c, despite the intratumor heterogeneity between T1b and T1c (Fig 2B bottom-right). Again, under sorafenib treatment (T2), 1/1 mutation in T2a and 2/3 mutations in T2b were captured in the cfDNA (Fig 2B bottom-right). The *CTNNB1* A45_E54del mutation that was detected in T1c but no longer detectable in T2b was detected in the cfDNA, suggesting the mutation remained in the tumor. We observed a moderate correlation between the VAFs in the cfDNA and in the tumors (*R*^2^ = 0.44, *P* < .01, Data Supplement).

Taken together, our results highlight the ability of cfDNA to capture the mutations of the independent primary tumors in multicentric HCC.

### cfDNA Captures the Intratumor Heterogeneity of Intrahepatic Metastatic HCC

**FIG 3.**
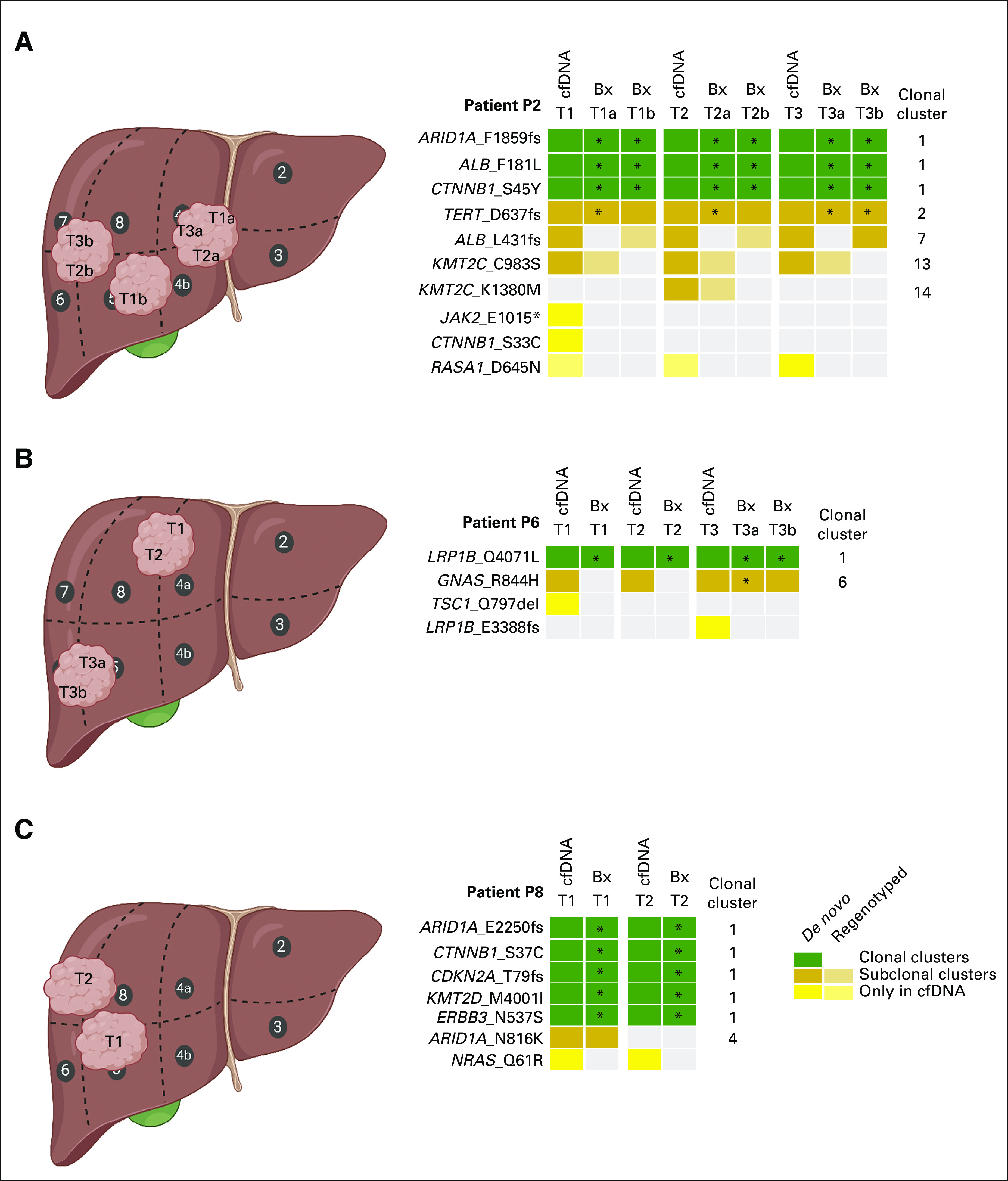
cfDNA captures the intratumor heterogeneity of intrahepatic metastatic HCC. Schematic representation of three patients, (A) P2, (B) P6, and (C) P8, with intrahepatic metastatic HCC showing the location of the biopsies taken (left) and the variants identified in biopsies and cfDNA in the regions covered by the targeted sequencing panel (numbered according to the clonal clusters shown in the Data Supplement). Mutations in the clonal and subclonal clusters are indicated in green and orange, respectively, further indicated as de novo (dark) or regenotyped (light). Mutations detected only in the cfDNA are indicated with a yellow box. Mutations including * symbols indicate those detected as clonal. Note that the location for P6 T1a/T1b is not well defined. Created with BioRender.com. cfDNA, cell-free DNA; HCC, hepatocellular carcinoma; P, patient.

WES analysis indicates that the remaining five patients had genetically related tumors that constitute intrahepatic metastases (Data Supplement). To evaluate the capacity of cfDNA to detect clonal and subclonal mutations in IM HCCs undergoing systemic therapy, we performed clonal reconstruction of the biopsies using PhylogicNDT^20^ and compared the mutations detected in the cfDNA to the clonal composition. Consistent with the characteristic of IM HCC, all biopsied tumors in P2, P6, P7, P8, and P9 shared the clonal cluster (cluster 1, containing mutations such as *CTNNB1* S45Y and *ARID1A* F1859fs in P2; *LRP1B* Q4071L in P6; and *CTNNB1* S37C and *CDKN2A* T79fs in P8, Data Supplement). In all patients, cfDNA consistently captured all mutations in the clonal cluster, except for P9 T1 cfDNA for which sequencing depth was low (Fig 3, Data Supplement).

Intratumor genetic heterogeneity was evident in all five IM cases, including four cases (P2, P6, P8, and P9) in which at least one subclone harbored mutations targeted by our cfDNA assay. Across the four patients, 15/18 (83%) of the mutations in the nonclonal clusters were detected in the cfDNA at the corresponding time (Fig 3, Data Supplement). In P2 and P8, the cfDNA samples completely recapitulated the pattern of clonal and subclonal mutations over the clinical course. In P2, the tumor nodule in segment 4 (T1a/T2a/T3a) harbored a subclonal *KMT2C* C983S mutation (cluster 13) and a subclonal *KMT2C* K1380M mutation (cluster 14) in T2a, whereas the tumor nodules in segments 5/6/7 (T1b/T2b/T3b) harbored a subclonal *ALB* L431fs mutation (cluster 7; Fig 3A). The cfDNA captured the intratumor heterogeneity at the corresponding time points. In P8, the *ARID1A* N816K (cluster 4) mutation was detected in the pretreatment cfDNA and biopsy in segment 5, but not detected in the cfDNA and segment 7 biopsy taken during sorafenib treatment (Fig 3C), suggesting the loss of the subclone harboring this variant after treatment. By contrast, in P6, a population containing the *GNAS* R844H mutation (cluster 6) was not identified at T1 pretreatment and T2 during sorafenib, but was detected following treatment (T3a and T3b), which could have led to the incorrect conclusion that the mutation was acquired during treatment (Fig 3B). However, the *GNAS* R844H mutation was captured in all cfDNA samples during the clinical course of the patient, including the pretreatment, suggesting this mutation was, in fact, not captured in the tumor biopsies because of tumor heterogeneity. In all five IM HCCs, we observed significant correlation between the VAF in cfDNA and the VAF detected in the tumor biopsies (*R*^2^ between 0.14 and 0.55, all *P* < .05, Data Supplement). We also observed that the changes in the VAF in the cfDNA mirrored the temporal changes in the cfDNA concentration (Data Supplement).

Together, our results suggest that cfDNA captures intratumor heterogeneity, including all clonal mutations and a substantial fraction of subclonal mutations, in IM HCCs.

### Serial cfDNA Captures More Mutations Than Single Diagnostic Biopsy

**FIG 4.**
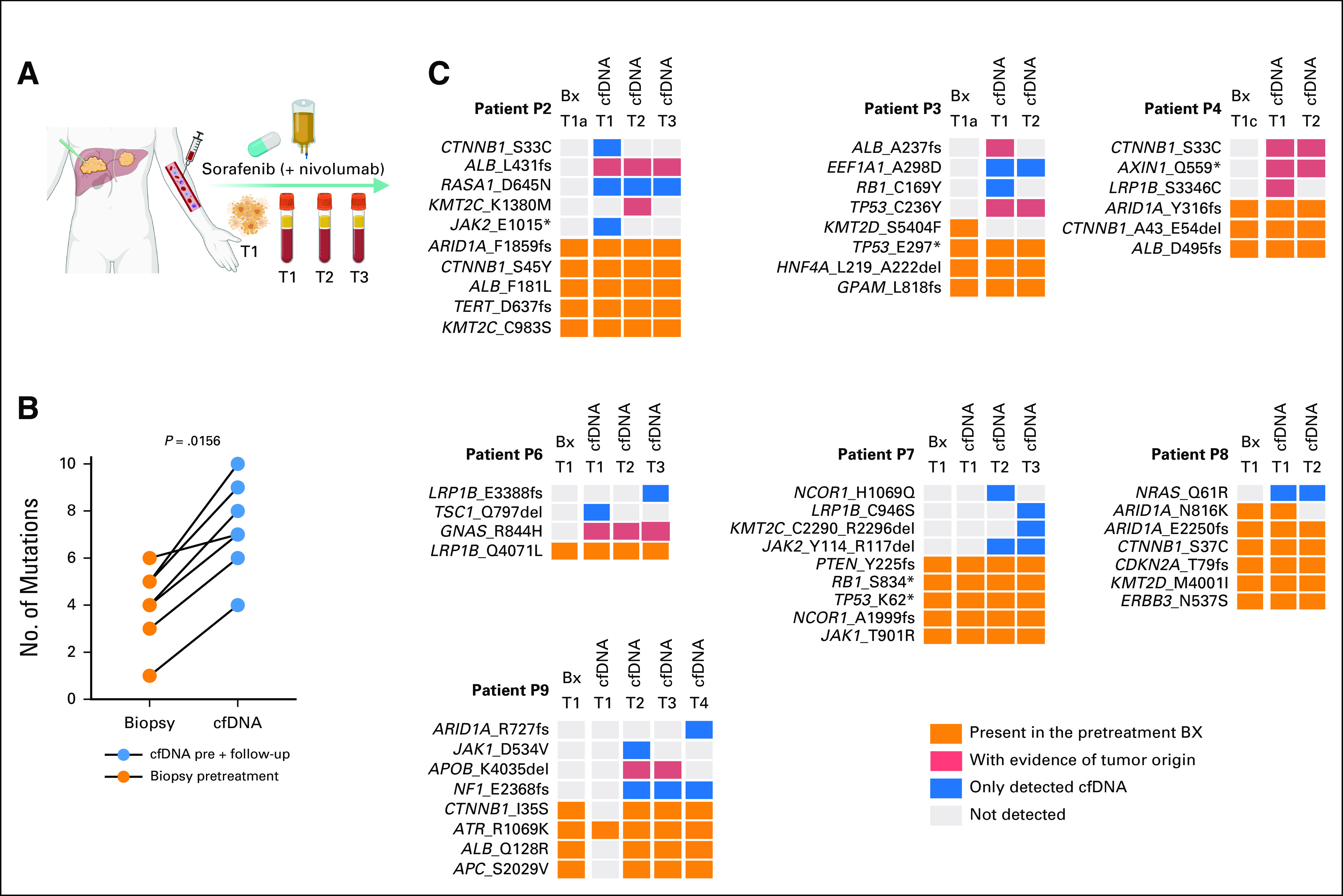
Serial cfDNA captures more mutations than single diagnostic biopsy. (A) Schematic representation comparing pretreatment biopsy with cfDNA obtained during patients' follow-up. (B) Line chart showing the number of nonsynonymous mutations detected in any cfDNA (blue) compared with the pretreatment tumor biopsy (orange). Statistical comparison was performed using a Wilcoxon signed-rank test. (C) Nonsynonymous mutations detected in pretreatment tumor biopsies (N = 7) and plasma cfDNA (N = 19). Samples are grouped by patient. Samples at T1 were taken synchronously. Orange boxes indicate mutations detected in the pretreatment biopsy; light red boxes indicate mutations absent in the pretreatment biopsy, detected in the cfDNA, but with a confirmed liver tumor origin; blue boxes indicate mutations detected only in the cfDNA; and gray boxes indicate the absence of the mutation. Created with BioRender.com. cfDNA, cell-free DNA; P, patient.

To simulate the clinical scenario where a single diagnostic tumor biopsy is performed or a single tumor area from a resection is used for genetic profiling to guide treatment decisions, we compared the mutations identified in one pretreatment tumor biopsy (selected on the basis of the highest number of detected mutations) with those identified in the cfDNA samples obtained from the matched patients (Fig 4A). Although we did not detect more mutations in the pretreatment cfDNA samples compared with the pretreatment biopsies (*P* > .05, Wilcoxon signed-rank test; Data Supplement), we identified a significantly higher number of mutations in the serial cfDNA samples compared with the pretreatment biopsy (*P* = .0156, Wilcoxon signed-rank test; Fig 4B). Altogether, 24 unique variants were detected in the cfDNA but not in the pretreatment biopsies, including 9 (38%) with evidence of tumor origin (ie, detected in at least one subsequent tumor biopsy, Fig 4C). A comparison of the mutations detected in the pretreatment cfDNA to those in the selected pretreatment biopsy revealed a median of 2 additional mutations (range, 0-4) detected in the cfDNA that were absent from the pretreatment tumor biopsies (Fig 4C). These 14 unique variants include six that were present in other pretreatment biopsies (ie, intratumor heterogeneity at diagnosis) and one that was observed in tumor biopsies only during follow-up. Two of the variants of unknown origin (ie, no evidence of tumor origin) are clinically significant, with the *NRAS* Q61R mutation (P8) being a standard-of-care biomarker to predict resistance to anti–estimated glomerular filtration rate therapy in colorectal carcinoma (level R1 in OncoKB^21^) and the *TSC1* Q797del (P6), a standard-of-care biomarker to predict response to everolimus (level 1 in OncoKB^21^). Compared with the pretreatment cfDNA samples, 11 mutations were only detected in the follow-up cfDNA obtained during or after treatment (Fig 4C), including two that were detected in the follow-up tumor samples but not in the pretreatment tumor biopsies, demonstrating the capacity of cfDNA to capture emerging mutations because of treatment-specific selective pressures.

Taken together, our results demonstrate the capacity of serial cfDNA collections to detect intratumor heterogeneity and tumor evolution compared with a single diagnostic biopsy.

## DISCUSSION

HCC can often be diagnosed without liver biopsy, limiting access to tumor tissues for genetic profiling. Only in rare cases when the clinical suspicion for HCC cannot be confirmed by imaging is a liver biopsy recommended.^22^ However, the utility of liver biopsies in genetic profiling has been questioned, owing to intratumor (or interlesion, in multicentric HCCs) genetic heterogeneity and the challenges associated with biopsies of small tumors.^23,24^ Recent studies performing multiregion sequencing of tumor^8,25,26^ or multiple nodules within the same patient^7,8^ demonstrated that the extent of interlesion and intratumor heterogeneity in HCC cannot be characterized by analyzing a single lesion. Here, we found that cfDNA better captures the heterogeneity of multicentric and intrahepatic metastatic HCC compared with single liver biopsy.

Multinodular HCC accounts for up to 70% of HCCs.^4^ Although the precise fraction of multicentric HCCs is unknown, two studies reported that 56%-71% of multiple liver lesions constitute multicentric HCCs,^27,28^ suggesting that MC is rather common. Here, we demonstrated that in our two multicentric HCCs, mutations from genetically independent tumors were captured by the cfDNA. Given the better prognosis in patients with multicentric than IM HCCs,^29,30^ being able to distinguish them without the need for multiple biopsies would have important clinical implications. One could hypothesize that multimodal VAF distribution in the cfDNA, the presence of multiple mutations in a single cancer driver gene at different VAFs, or mutations known to be mutually exclusive may suggest multicentricity. We observed these features in P3, in which the mutations detected in the biopsies T1b and T2a (including *TP53* C236Y) were at < 5% VAF in the cfDNA and the mutations detected in the biopsies T1a and T2b (including *TP53* E297*) were at > 10% VAF in the cfDNA (Data Supplement). By contrast, we did not observe multimodal distributions in the P4 (multicentric) cfDNA but we did in P7 (IM), which may be due to the presence of subclonal populations. Although it is unlikely that multicentric and IM HCCs can be unambiguously distinguished using cfDNA alone, with larger sequencing panels, cfDNA may suggest the presence of IM HCCs in some cases.

In four of the IM HCCs, we showed that all mutations clonal across all tumor biopsies were detected in the cfDNA, along with > 80% of the mutations outside of the clonal clusters, in line with reports in other solid cancer.^31,32^ The capacity for cfDNA to detect clonal and subclonal variants suggests that it may be useful for monitoring the appearance of variants emerging or lost during systemic treatment. Unfortunately, the size of our cohort together and the fact that no genetic variant in our gene panel has been associated with the response to sorafenib and nivolumab in patients with HCC do not allow us to make any conclusion regarding the use of cfDNA to detect markers of resistance to these therapies. We also note that the VAFs of the mutations identified in the cfDNA did not always correspond to treatment response. We speculate this may be due to the difference in timing between cfDNA sampling and response assessment, where the observed cfDNA changes may result from transient response or bursts of apoptosis, but may also be explained by differential response and tumor shedding between nodules over time. Nonetheless, compared with a single diagnostic biopsy, we have identified two mutations that were only detected in the follow-up tumor samples but not in the pretreatment tumor biopsies. Furthermore, a single cfDNA sample may not capture all driver mutations but serial cfDNA sampling would maximize capturing the temporal heterogeneity of driver mutations (eg, *CTNNB1* S33C in P2, *ALB* A237fs in P3, and *ARID1A* R727fs in P9). Our results demonstrate that serial cfDNA samples can capture tumor evolution over the course of treatment.

Intriguingly, 20% of the mutations detected in cfDNA via targeted sequencing could not be detected in any tumor biopsy by WES, even when several biopsies from different locations were analyzed. These cfDNA-specific mutations include likely driver mutations, such as *CTNNB1* S33C, *ARID1A* R727fs, and *NF1* E2368fs. They also include *TSC1* Q797del and *NRAS* Q61R, both standard-of-care biomarkers of response to targeted therapy in other solid cancers. In a previous study in colorectal carcinoma, Parikh et al^31^ also showed that liquid biopsies enable the identification of clinically relevant resistance alterations not detected in the matched tumor biopsy samples. Given the interlesion and intratumor heterogeneity, one could hypothesize that some of these cfDNA-specific mutations may have derived from unsampled tumor nodules or even tumors outside of the liver, related or unrelated to the current HCC(s). Indeed, except for P4, not all liver lesions were biopsied, and P1 had a radiologically diagnosed extrahepatic intraductal papillary mucinous neoplasm (Data Supplement). Thus, it is possible that some of the cfDNA-specific mutations originated from these unsampled lesions. Moreover, the detection limits for the UMI-tagged targeted sequencing assay and WES are approximately 0.1% and 1%-2%, respectively. Coupled with the increased sequencing depth of the targeted sequencing assay (median 1930× *v* 145× for WES), one could argue that at least some of the cfDNA-specific mutations may have been present at levels below the detection limit of WES in the tumors. Finally, given the age of the patients, it is also possible that some mutations detected in the cfDNA may result from clonal hematopoiesis, although we sequenced the matched peripheral blood mononuclear cells with the same assay. In fact, several of the cfDNA-specific mutations are in genes previously reported to be affected by clonal hematopoiesis (eg, *JAK2* and *KMT2C*).^33,34^

Although several studies have provided evidence of the utility of cfDNA as a surrogate sample for genetic profiling of HCC,^9,10^ also in the context of systemic treatment,^11^ this is the first study, to our knowledge, that demonstrates the capability of cfDNA to capture the high heterogeneity of multinodular HCC. Our results highlight the capacity of the cfDNA to detect mutations in multicentric HCCs, clonal and subclonal mutations present in the tissue, as well as other variants missed by a single liver biopsy.
